# Complete genome sequence of *Bacillus toyonensis* WHQ17, a bacteriocin-producing strain isolated from soil of Xiangshan Mountain

**DOI:** 10.1128/mra.00969-26

**Published:** 2026-09-18

**Authors:** Hualin Liu, Congyue Yang, Bingyue Xin

**Affiliations:** 1 Research & Development Division, Perfect (Guangdong) Co., Ltd., Zhongshan, Guangdong Province, China; 2 Research & Development Division, Perfect Life & Health Institute, Zhongshan, Guangdong Province, China; 3 Anhui Province Key Laboratory of Pollutant Sensitive Materials and Environmental Remediation, College of Life Sciences, Huaibei Normal University 58286, Huaibei, Anhui Province, China; DOE Joint Genome Institute, Berkeley, California, USA

**Keywords:** *Bacillus toyonensis*, complete genome, bacteriocins

## Abstract

*Bacillus toyonensis* WHQ17, isolated from Xiangshan Mountain soil, produces a bacteriocin with specific anti-*Bacillus cereus* activity. Here, we present its complete genome, which comprises one chromosome and five plasmids, and identify three putative bacteriocin biosynthetic gene clusters for future characterization.

## ANNOUNCEMENT

*Bacillus toyonensis* is a gram-positive, rod-shaped, facultatively anaerobic, spore-forming bacterium widely found in natural environments and animal gastrointestinal tracts (1). As a well-established probiotic, it has been safely used in animal feed for decades (2, 3). Recent studies have focused on its bacteriocins, such as the unmodified bacteriocin toyoncin and the circular bacteriocin toyoncin WJY28, which show potent, specific activity against foodborne pathogens and hold promise for food preservation (4, 5).

**Fig 1:**
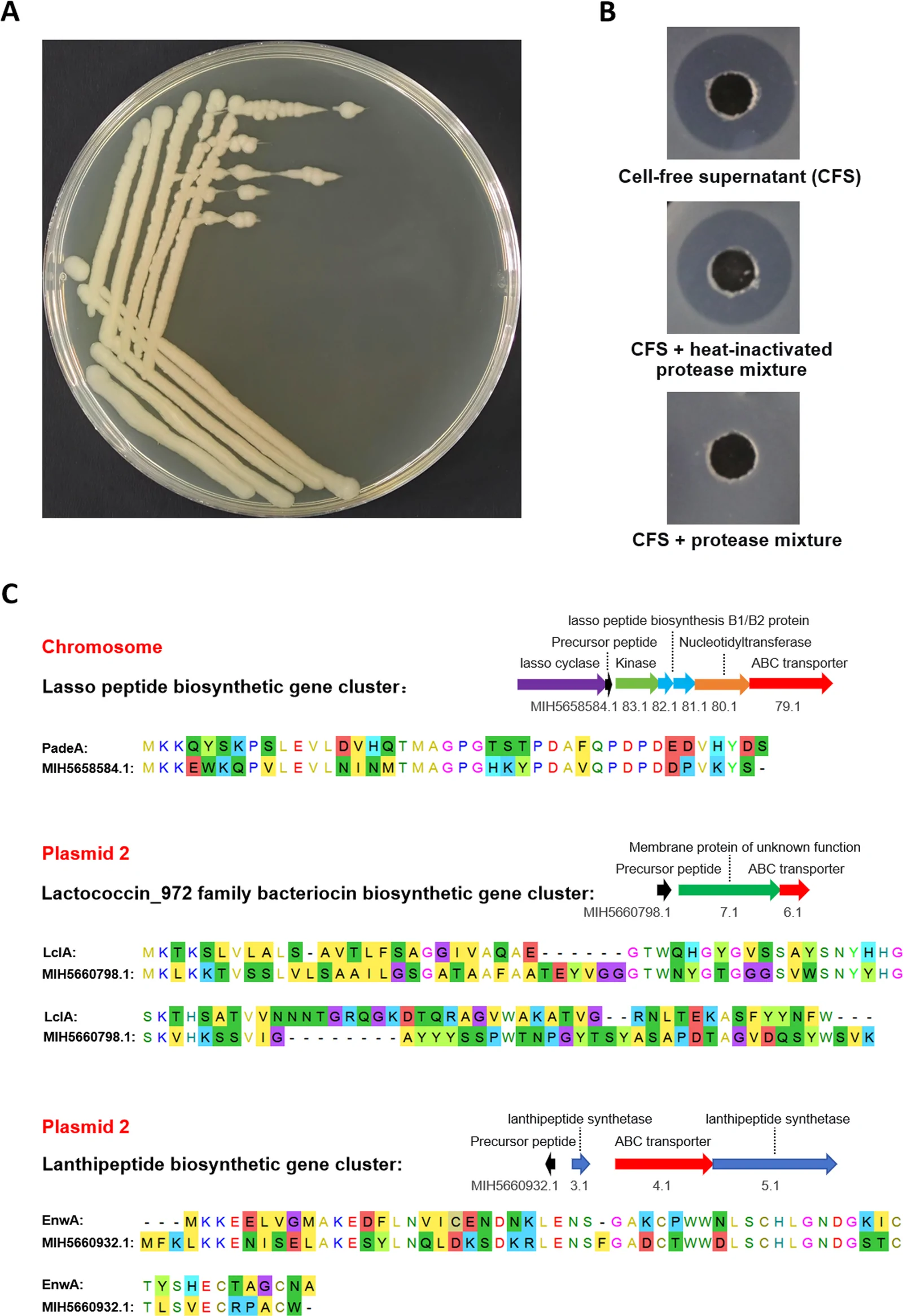
Characteristics of *Bacillus toyonensis* WHQ17. (**A**) Colony morphology on TSA plate after 24 h at 25°C. (**B**) Antimicrobial activity of cell-free supernatant (CFS) against *B. cereus* CMCC 63303 by the agar well diffusion method. CFS was obtained by culturing in TSB at 25°C for 8 h with shaking (200 rpm), followed by centrifugation (12,000 rpm, 10 min). Treatments shown are untreated CFS, CFS with heat-inactivated protease mixture, and CFS with active protease mixture. Protease treatment abolished the inhibition zone, indicating that the antimicrobial substance belongs to bacteriocins. (**C**) Predicted bacteriocin biosynthetic gene clusters (BGCs) (lassopeptide, Lactococcin_972 family, and lanthipeptide) and sequence alignment of their precursor peptides with the most similar known bacteriocins: PadA (paeninodin), LclA (Lactococcin_972), and EnwA (enterocin W α-subunit), respectively.

Strain WHQ17 was isolated from Xiangshan National Forest Park soil, Huaibei, China (33.99°N, 116.81°E), by serial dilution and spread plating on tryptic soy agar at 25°C for 24 h; a single colony was purified (Fig. 1A). Antimicrobial assays by the agar well diffusion method (4) revealed that WHQ17 produces a highly specific anti-*Bacillus cereus* substance, and its activity was inactivated by protease treatment (α-chymotrypsin and trypsin, 1 mg/mL each), confirming its bacteriocin nature (Fig. 1B).

Genomic DNA was extracted from overnight TSB cultures with the TaKaRa MiniBEST Kit v3.0, yielding ~30 µg; 1 μg was used for Illumina and 5 μg for Oxford Nanopore Technology (ONT) libraries. For ONT sequencing, the extracted genomic DNA was used directly without shearing or size selection. Libraries were prepared with the SQK-RBK004 Rapid Barcoding Kit and sequenced on a MinION device (FLO-MIN106D flow cell). Base calling with Guppy v6.2.1 generated 156,653 reads (N50, 18,327 bp; total, 2.183 Gb). For Illumina sequencing, genomic DNA was sheared with a Covaris LE220R-plus instrument, followed by size selection using AMPure XP magnetic beads (no dedicated size-selection instrument was used). Libraries were constructed using the NEBNext Ultra II DNA Library Prep Kit and sequenced on a NovaSeq 6000 platform (S4 Reagent Kit), generating 150 bp paired-end raw reads. Raw reads were preprocessed with fastp v1.3.6 (6), yielding 8,457,250 clean reads (1.268 Gb). Hybrid assembly with Unicycler v0.4.9 (7) was followed by polishing with Racon v1.4.20 (8) and Pilon v1.24 (9). All tools were run with default parameters unless otherwise specified.

The final assembled genome comprised six circular replicons: one chromosome and five plasmids. Circularity was assessed by Unicycler via closed-loop paths; the chromosome was rotated to start at the putative *dnaA* gene, whereas the plasmids were not rotated owing to the absence of *repA* homologs. Annotation using PGAP v6.11 (10) predicted 6,303 protein-coding genes, 106 tRNAs, and 42 rRNAs (Table 1). dDDH and ANI values of 86.80% and 98.65%, respectively, between WHQ17 and the type strain *B. toyonensis* P18 (GCF_016605985.1) far exceeded the species thresholds (70% for dDDH and 96% for ANI) (11), as calculated with the GGDC 3.0 (12) and EzBioCloud ANI Calculator (13), confirming that WHQ17 belongs to *B. toyonensis*. BAGEL4 (14) (default parameters) and antiSMASH (15) (relaxed mode) analyses predicted three putative bacteriocin biosynthetic gene clusters, corresponding to a lasso peptide, a Lactococcin_972 family bacteriocin, and a lanthipeptide (Fig. 1C). The predicted precursor peptides show low similarity to their closest known homologs and likely account for the observed antimicrobial activity. Future studies will focus on identifying these bacteriocins and evaluating their potential as natural preservatives against *B. cereus*.

**TABLE 1:** Genome features of *Bacillus toyonensis* WHQ17

Segment	Length (bp)	G + C content (%)	No. of genes	No. of tRNAs	No. of rRNAs	No. and types of bacteriocin BGCs	Accession number
Chromosome	5,333,284	35.49	5,506	106	42	1 lasso peptide	JCBIIR010000001.1
Plasmid 1	501,947	32.97	469	0	0	0	JCBIIR010000002.1
Plasmid 2	266,359	32.56	248	0	0	1 Lactococcin_972 family bacteriocin + 1 lanthipeptide	JCBIIR010000003.1
Plasmid 3	51,457	33.71	57	0	0	0	JCBIIR010000004.1
Plasmid 4	10,517	30.63	12	0	0	0	JCBIIR010000005.1
Plasmid 5	10,182	27.86	11	0	0	0	JCBIIR010000006.1
Total	6,173,746	35.12	6,303	106	42	3	JCBIIR000000000.1

## Data Availability

The genome sequence of *Bacillus toyonensis* WHQ17 is available in GenBank under the master accession number JCBIIR000000000.1 (Table 1). The raw sequences are available under Sequence Read Archive (SRA) accession numbers SRX34668026 (Illumina) and SRX34668027 (ONT).
